# Node Depth Adjustment Based Target Tracking in UWSNs Using Improved Harmony Search

**DOI:** 10.3390/s17122807

**Published:** 2017-12-04

**Authors:** Meiqin Liu, Duo Zhang, Senlin Zhang, Qunfei Zhang

**Affiliations:** 1 State Key Laboratory of Industrial Control Technology, Zhejiang University, Hangzhou 310027, China; 2 College of Electrical Engineering, Zhejiang University, Hangzhou 310027, China; zhangduo@zju.edu.cn (D.Z.); slzhang@zju.edu.cn (S.Z.); 3 School of Marine Science and Technology, Northwestern Polytechnical University, Xi’an 710072, China; zhangqf@nwpu.edu.cn

**Keywords:** target tracking, node depth adjustment, improved harmony search, underwater wireless sensor networks

## Abstract

Underwater wireless sensor networks (UWSNs) can provide a promising solution to underwater target tracking. Due to the limited computation and bandwidth resources, only a small part of nodes are selected to track the target at each interval. How to improve tracking accuracy with a small number of nodes is a key problem. In recent years, a node depth adjustment system has been developed and applied to issues of network deployment and routing protocol. As far as we know, all existing tracking schemes keep underwater nodes static or moving with water flow, and node depth adjustment has not been utilized for underwater target tracking yet. This paper studies node depth adjustment method for target tracking in UWSNs. Firstly, since a Fisher Information Matrix (FIM) can quantify the estimation accuracy, its relation to node depth is derived as a metric. Secondly, we formulate the node depth adjustment as an optimization problem to determine moving depth of activated node, under the constraint of moving range, the value of FIM is used as objective function, which is aimed to be minimized over moving distance of nodes. Thirdly, to efficiently solve the optimization problem, an improved Harmony Search (HS) algorithm is proposed, in which the generating probability is modified to improve searching speed and accuracy. Finally, simulation results are presented to verify performance of our scheme.

## 1. Introduction

Over 70% of the earth’s surface is covered by oceans and rivers. Underwater research has great potential due to a lot of undeveloped resources. In recent years, underwater wireless sensor networks (UWSNs) have gradually become an important technology for human beings to explore and utilize underwater resources. As an extension of terrestrial wireless sensor networks, UWSNs can be used for disaster warning, environmental monitoring, underwater target detection and tracking [1,2,3,4,5]. In this paper, we focus on the problem of underwater target tracking using UWSNs.

UWSNs can provide a reliable solution to underwater target tracking. For moving underwater targets, UWSNs with high-density sensing ability are needed to collect measurements and transmit estimated data. Most research about target tracking in UWSNs is based on densely and statically deployed underwater nodes. However, due to the high cost of underwater sensor nodes and complex underwater environments, we have to sparsely deploy nodes in practical 3D underwater areas [6,7]. In addition, the deployment work of nodes is always completed before the network is put into service, and nodes will not change their position after being put into water. Such a network can achieve an accurate tracking performance when the target moves close enough to it, but if a target is detected in an area with a low coverage rate, the network will fail to track it accurately, and it lacks the adaptive adjustment ability to dynamic events. Therefore, we need to improve the flexible sensing and communication ability of UWSNs.

As we know, underwater nodes are floating underwater with the help of buoys and mooring lines. In this paper, we consider that the nodes are equipped with a node depth adjustment system. Detweiler et al. [8] presented a depth adjustment system that connects to underwater nodes. Wu et al. proposed a depth adjustment scheme to maximize the coverage in 3D space [9]. The depth adjustment for underwater nodes is always applied to issues of network deployment and routing protocol. It can improve the network coverage rate and data packet delivery ratio, thus increasing network reliability [10,11]. Inspired by these research results, this paper proposes a novel scheme to improve target tracking accuracy. It is assumed that nodes are located at their original position when they are not working. Once some nodes are woken up by fusion center, they adjust their depth according to commands from the fusion center and sense the target at the optimal depth, in order to improve tracking performance. Thus, the key problem is how to determine optimal depth of nodes during the tracking task, which can be converted to a dynamic optimization problem under the constraint of moving range. In our previous work, we provided some solutions to select the optimal node cluster for target tracking [12,13]. Fisher Information Matrix (FIM) and its inverse matrix posterior Cramer Rao Low Bound (PCRLB) can reflect estimation accuracy, and we employed them as criteria to select the optimal node cluster. Hence, we will take FIM as an objective function in this paper and compute the optimal nodes’ depth in the framework of optimization problem.

As an optimization problem, finding the global optimal analytical solution becomes extremely difficult. In theory, the optimal depth can be determined if we discretize the constraint moving range and perform an exhaustive search. However, this method is not practical because of the heavy computational burden. For this reason, an improved Harmony Search algorithm (HS) is proposed to solve this optimization problem. HS is a meta-heuristic optimization algorithm that mimics the improvisation process of music players. It is a population-based search algorithm that can successfully solve optimization problems [14,15,16]. In this paper, each harmony represents nodes’ moving distance, which is related to final position of nodes. For traditional HS, a harmony memory matrix is randomly initialized within moving range. Some harmonies may lead a node’s final position to be far from the target; however, such harmonies are not that useful. The closer the distance between node and target, the better the performance may be. Therefore, we adjust the probability of a new harmony being generated as increasing as the distance to the target decreases, in order to improve tracking accuracy and searching speed.

The main contributions of this paper are threefold. Firstly, we propose a node depth adjustment scheme to dynamically improve tracking accuracy in UWSNs. This is a novel idea that has not been done before. Secondly, we employ the relationship between FIM and nodes’ depth as an objective function, and determine moving distance in the framework of optimization problems. Thirdly, an improved HS is proposed to solve this optimization problem, improve tracking accuracy and searching speed.

The rest of the paper is organized as follows. In Section 2, an overview of the related work about this paper is provided for readers. In Section 3, the target tracking problem in UWSNs is formulated based on node depth adjustment. In Section 4, the relationship between FIM and nodes’ depth is employed to determine nodes’ depth, and an optimization optimization problem is formulated. An improved Harmony Search algorithm is proposed to solve the optimization problem in Section 5. Simulation results are presented in Section 6 and conclusions of this paper are drawn in Section 7.

## 2. Related Work

As for the development of UWSNs, the research of target tracking in UWSNs has gradually become an important issue. Huang et al. [17] presented a two cluster-based distributed particle filter tracking algorithms. The first algorithm focused on tracking accuracy, while the second considered the balance between consumption and tracking accuracy. Wang et al. combined a particle filter with an interacting multiple model to solve maneuvering target tracking in UWSNs [18]. Wali et al. proposed and scrutinized two three-dimensional cluster-based UWSN architectures for identifying and tracking submerged moving intruders in [19]. The communication links from the intruder sensing nodes to the base station were based on radio frequency. As we know, energy consumption is also a key problem for target tracking UWSNs. To save energy consumption in UWSNs, Yu et al. [20] proposed a wake-up/sleep scheme based underwater node selection algorithm to track targets efficiently. In [21], Zhang et al. proposed a tracking protocol considering energy consumptions in both the target and sensor nodes. The protocol was designed in two aspects. The first was the passive listening mechanism and duty-cycle strategy for targets and the second was detection-based ranging packet transmission for sensor nodes.

To save on communication costs during the tracking process, we proposed an artificial measurement based energy-efficient filter that implements the trade-off between communication costs and tracking accuracy [22]. The sensor nodes would not send weak measurement to the fusion center. Instead, an artificial measurement would be generated in the fusion center to guarantee tracking accuracy. As shown in our previous work, we found that the geometry between nodes and target had a big effect on the accuracy of target tracking in UWSNs. Since a nonlinear measurement model was always utilized, the locations where distance measurements were collected had a profound effect on the tracking accuracy. When sensing nodes were close to each other, the measurements provided by them were similar, and we could not get enough useful information. However, when nodes were in different directions of targets, they could provide different information and improve tracking accuracy. Based on the effect, we designed several node selection algorithms to wake the best node combination for tracking tasks [12,13,23,24]. We quantized the effect of geometry based on a posterior Cramer–Rao lower bound (PCRLB) and minimized PCRLB to get the best nodes.

In the aforementioned results, the sensor nodes were considered static underwater. However, with static underwater nodes, a lot of nodes should be deployed to guarantee tracking accuracy. It was expensive and impractical to achieve such a network. In [25], Moreno-Salinas et al. utilized sea surface deployed UWSNs to position underwater targets. An optimal node placement for underwater target positioning was proposed using the Fisher Information Matrix. However, the nodes were static after placement, which was not practical for tracking a moving target. In [8], an autonomous depth adjust system was designed and applied to UWSNs by Detweiler et al. A winch based module was added to underwater nodes to enable depth adjustment and improve global sensing and communication ability. A lot of experiments showed great promise of the depth adjustment system. Based on node depth adjustment, Wu et al. proposed a routing protocol for UWSNs to improve the data packet delivery ratio in UWSNs [10]. It switched topology of the network through depth adjustment of the void nodes. In addition, Jiang et al. proposed a depth adjustment based deployment algorithm in UWSNs using a two-dimensional convex Hull and spanning tree, improving network connectivity and coverage [11]. The depth adjustment strategy based on time markers was used to achieve the three-dimensional overall network deployment. However, node depth adjustment for target tracking has not been found in literature.

Inspired by the effect of node geometry and node depth adjustment, we firstly propose using node depth adjustment to improve tracking accuracy. This paper considers a dynamical node depth adjustment scheme that moves nodes to the optimal depth for tracking the target. We derive the relationship between node depth and FIM, and utilize the relationship as a metric to determine movement of modes. Thus, the key problem is converted to how to find the optimal depth under the framework of optimization problem. In [26], Yang et al. proposed an optimal coordination strategy for sensor motion to maximize the tracking accuracy and the search space was reduced to improve efficiency. A harmony search based deployment algorithm was proposed by Mohd Alia et al. that can locate the optimal number of sensor nodes as well as their optimal locations for maximizing the network coverage and minimizing the network cost in [27]. In this paper, we utilized the harmony search algorithm to solve the optimization problem of node depth. The harmony search is modified to improve searching performance according to target position.

## 3. Problem Formulation

This section formulates the problem of single target tracking in UWSNs. The issues to be covered include network model, target state model, measurement model and multi-sensor Particle Filter (PF) for target tracking.

### 3.1. Network Model

**Figure 1:**
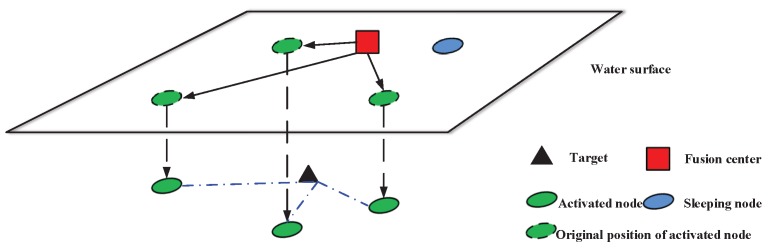
Network model (the black triangle is a moving target; the red square is the fusion center; the blue oval is the sleeping node, which is static at the water surface; the green ovals are activated nodes, which move down vertically for sensing the target according to commands from the fusion center).

In real situations, the sensors cannot be always placed at will, either due to physical or mission constraints. Thus, we refer to the scenario in Ref. [25], and we tackle the case where the sensors are floating in the sea surface at first. If a moving target is within the sensing range, the fusion center will wake up nodes to participate in the tracking task, and send commands to activated nodes to make them move vertically to the optimal depth. Then, the nodes will sense the target and send measurements back to the fusion center. As shown in Figure 1, the black triangle is a moving target; the red square is fusion center; the blue oval is a sleeping node, which is static at the water surface; the green ovals are activated nodes, which move vertically to the optimal depth for sensing the target. It should be noted that the moving range of nodes can be modified according to target’ position. Otherwise, it remains dormant for energy efficiency. After receiving measurements from all selected nodes, the fusion center will estimate the target state via a tracking algorithm and communicate with the base station by radio signal.

### 3.2. Target State Model

In this paper, the target is assumed to be a slowly maneuvering point that moves at a known depth *h*. For the sake of simplicity, the nearly constant turn (CT) model can be utilized. It should be noted that our scheme is not limited to the CV model; for some complex motion models, the interacting multiple model (IMM) approach can be combined with our scheme. Thus the target state is given by following equation:

(1) $$x_{k+1}=F_{k}x_{k}+w_{k},$$

where the target state at time *k* is given by $x_{k}={\left[x,v_{x},y,v_{y}\right]}^{T}$. (x,y) is the target’s location; while $v_{x}$ and $v_{y}$ are the corresponding velocity in *x* and *y* coordinates, respectively. $F_{k}$ is the state transition matrix; the process noise $w_{k}$ is always assumed to be Gaussian with zero mean and covariance matrix $Q_{k}$. The state transition matrix $F_{k}$ and process noise covariance matrix $Q_{k}$ are given as follows:

(2) $$F_{k}=\left[\begin{array}{cccc} 1 & \frac{\sin wT}{w} & 0 & -\frac{1-\cos wT}{w}\\ 0 & \cos wT & 0 & -\sin wT\\ 0 & \frac{1-\cos wT}{w} & 1 & \frac{\sin wT}{w}\\ 0 & \sin wT & 0 & \cos wT \end{array}\right],$$

(3) $$Q_{k}=q^{2}\left[\begin{array}{cccc} \frac{2(wT-\sin wT)}{w^{3}} & \frac{1-\cos wT}{w^{2}} & 0 & \frac{wT-\sin wT}{w^{2}}\\ \frac{1-\cos wT}{w^{2}} & T & -\frac{wT-\sin wT}{w^{2}} & 0\\ 0 & -\frac{wT-\sin wT}{w^{2}} & \frac{2(wT-\sin wT)}{w^{3}} & \frac{1-\cos wT}{w^{2}}\\ \frac{wT-\sin wT}{w^{2}} & 0 & \frac{1-\cos wT}{w^{2}} & T \end{array}\right],$$

where *T* is the sampling interval, *w* is turn rate, which is known and constant, and *q* is the process parameter.

### 3.3. Measurement Model

Each sensor node in UWSNs is equipped with a wireless acoustic sensor. The measurement model for the acoustic sensor contains a base frequency measurement model (narrow-band processing), an acoustic spectrum pattern model (narrow-band processing), and an acoustic sound pressure measurement model (wide-band processing) [28,29]. In this paper, sensors measure distance to the target via transmitting acoustic pulses and calculate time of arrival (TOA) from the pulse to echoes. The measurement model of the sensor $s_{th}$ at time *k* is given by

(4) $$Z_{k+1}^{s}=h_{k+1}^{s}\left(x_{k+1},x^{s},y^{s},z^{s}\right)+v_{k+1}^{s},$$

(5) $$\begin{array}{c} h_{k+1}^{s}\left(\cdot \right)=\sqrt{{\left(x_{k+1}^{1}-x^{s}\right)}^{2}+{\left(x_{k+1}^{3}-y^{s}\right)}^{2}+{\left(h-z^{s}\right)}^{2}}, \end{array}$$

where $x_{k+1}^{1}$ and $x_{k+1}^{3}$ are the first and third elements of target state vector $x_{k+1}$, which represents the position of target at time k+1; $\left(x^{s},y^{s},z^{s}\right)$ is the location of sensor *s*; $h_{k+1}^{s}\left(x_{k+1},x_{k+1}^{s},y_{k+1}^{s},z_{k+1}^{s}\right)$ is the measurement function; $v_{k+1}^{s}$ is the measurement noise, which is assumed to be independent across time steps and across sensors, and follows a Gaussian distribution with parameters N(0,R).

### 3.4. Multi-Sensor Particle Filter for Target Tracking in UWSNs

The Particle Filter (PF) is an efficient way to solve nonlinear and non-Gaussian problems. Readers can refer to references [30,31] for detailed PF. In this paper, multi-sensor PF will be utilized for tracking issues.

It is assumed that $N_{s}$ sensing nodes take part in tracking the underwater target and transmit their measurements to the fusion center at each time step. Once receiving measurements vector $Z_{k+1}={\left[Z_{k+1}^{1},\cdots ,Z_{k+1}^{N_{s}}\right]}^{T}$, the fusion center merges measurements into a single multi-sensor measurement likelihood. The measurement likelihood over all $N_{s}$ nodes is given by

(6) $$p\left(Z_{k+1}|x_{k+1}^{i}\right)=\prod_{s}^{N_{s}}p\left(Z_{k+1}^{s}|x_{k+1}^{i}\right),$$

where

(7) $$p\left(Z_{k+1}^{s}|x_{k+1}^{i}\right)=\frac{\exp[-\frac{1}{2}{\left(Z_{k+1}^{s}-h_{k+1}^{s}\left(x_{k+1}^{i}\right)\right)}^{T}{\left(R\right)}^{-1}\left(Z_{k+1}^{s}-h_{k+1}^{s}\left(x_{k+1}^{i}\right)\right)]}{\sqrt{2\pi R}}$$

is probability density function (pdf) of the measurement likelihood regarding the measurement acquired by the $s_{th}$ sensing node at time k+1; $x_{k+1}^{i}$ is the *i*th particle at time k+1. Then, adopting the prior the transition prior $p(x_{k}|x_{k-1})$ as the proposal distribution, the importance weights of particles are calculated as:

(8) $$w_{k+1}^{i}=w_{k}^{i}p\left(Z_{k+1}|x_{k+1}^{i}\right).$$

## 4. Depth Adjustment Based Target Tracking in UWSNs

In this section, depth adjustment based tracking scheme is proposed. The relation between Fisher information matrix (FIM) and nodes’ depth is employed to determine the optimal depth, and an improved harmony search algorithm is utilized to solve the problem and improve searching speed.

### 4.1. Relationship between FIM and Node Depth

FIM is widely utilized in estimation and fusion issues, and it is the inverse matrix of PCRLB. Both of them stand for information contained in measurements [32,33,34]. During the tracking processing, the larger the FIM is, the more useful information we can get from measurements, and the better tracking performance we will achieve.

Therefore, to improve tracking accuracy via adjusting nodes’ depth, FIM can be utilized as a metric to determine the optimal depth. The FIM $J_{k+1}$ has the following definition:

(9) $$E\left[{\hat{x}}_{k+1}-x_{k+1}\right]{\left[{\hat{x}}_{k+1}-x_{k+1}\right]}^{T}\geq J_{k+1}^{-1}.$$

FIM $J_{k+1}$ is a the 4×4 matrix with elements

(10) $$J_{k+1}\left(i,j\right)=E_{p\left(M_{k+1,q},x_{k+1}\right)}\left[-\frac{\partial^{2}\log\;p\left(Z_{k+1}|x_{k+1}\right)}{\partial x_{k+1}\left(i\right)\partial x_{k+1}\left(j\right)}\right],$$

where $J_{k+1}\left(i,j\right)$ denotes the *i*th row and $j_{th}$ column element of $J_{k+1}$, $E_{p\left(M_{k+1,q},x_{k+1}\right)}$ denotes the expectation respect to $p\left(M_{k+1,q},x_{k+1}\right)$, and $x_{k+1}\left(i\right)$ denotes the *i*th element of vector $x_{k+1}$.

Since the measurements in vector $Z_{k+1}$ is Gaussian with nonzero mean, FIM $J_{k+1}$ can be calculated as follows [35]:

(11) $$J_{k+1}\left(i,j\right)={\left[\frac{\partial H_{k+1}\left(x_{k+1}\right)}{\partial x_{k+1}\left(i\right)}\right]}^{T}R^{-1}\left[\frac{\partial H_{k+1}\left(x_{k+1}\right)}{\partial x_{k+1}\left(j\right)}\right],$$

where $H_{k+1}\left(x_{k+1}\right)$ is the true distance vector of ${\left[h_{k+1}^{1},\cdots ,h_{k+1}^{N_{s}}\right]}^{T}$ in Equation (5). R is the measurement noise matrix. Consequently, we can get

(12) $$\begin{array}{cc} J_{k+1}= & \sum_{s=1}^{N_{s}}\left[\begin{array}{cc} \frac{{\left(x^{s}-x_{k+1}^{1}\right)}^{2}}{{h_{k+1}^{s}}^{2}R} & \frac{\left(x^{s}-x_{k+1}^{1}\right)\left(y^{s}-x_{k+1}^{3}\right)}{{h_{k+1}^{s}}^{2}R}\\ \frac{\left(y^{s}-x_{k+1}^{3}\right)\left(x^{s}-x_{k+1}^{1}\right)}{{h_{k+1}^{s}}^{2}R} & \frac{{\left(y^{s}-x_{k+1}^{3}\right)}^{2}}{{h_{k+1}^{s}}^{2}R} \end{array}\right]\\ & =\sum_{s=1}^{N_{s}}\left\{\frac{1}{[{\left(x_{k+1}^{1}-x^{s}\right)}^{2}+{\left(x_{k+1}^{3}-y^{s}\right)}^{2}+{\left(h-z^{s}\right)}^{2}]R}\left[\begin{array}{cc} {\left(x^{s}-x_{k+1}^{1}\right)}^{2} & \left(x^{s}-x_{k+1}^{1}\right)\left(y^{s}-x_{k+1}^{3}\right)\\ \left(y^{s}-x_{k+1}^{3}\right)\left(x^{s}-x_{k+1}^{1}\right) & {\left(y^{s}-x_{k+1}^{3}\right)}^{2} \end{array}\right]\right\}, \end{array}$$

where $h_{k+1}^{s}$ is the true distance from sensor $s_{th}$ to the target’s position, and it is calculated via Equation (5). It can be seen that the node’s depth $z^{s}$ is related to the value of $h_{k+1}^{s}$, and the term $h-z^{s}$ represents relative depth between node and target, which has much effect on the value of FIM. Since FIM is always used to present estimation accuracy, the value of FIM can be utilized as a metric to determine nodes’ depth. It should be noted that nodes will move before estimating target’s position at each time according to predicted FIM. To calculate FIM, the predicted target’s position $x_{k+1|k}$ will be considered in Equation (12) as target state.

### 4.2. Node Depth Adjustment Problem Formulation

Since relation between FIM and nodes’ depth has been introduced, this subsection formulates the optimization problem of adjusting node depth. The FIM is a matrix that is not easy to analyze; thus, the popular D-optimality criterion is adopted in this paper, and the determinant of FIM is calculated as the metric of tracking. The larger the determinant is, the more information we will get from measurements, and the better tracking performance will be achieved. In addition, the D-optimality criterion, the A-optimality and E-optimality criterion are also widely used in the optimization problem. They respectively minimize the trace and the largest eigenvalue of the PCRLB matrix. However, they are variant under scale changes in the parameters and linear transformations of the output, and it is not convenient to calculate PCRLB matrix [36,37]. Hence, the D-optimality criterion will be used in this paper.

We assume that there are $N_{s}$ nodes participating in tracking tasks at each time. The depth of sensor *s* is $z_{k}^{s}$ at time *k*. The sensor will move vertically downward, and its depth at time k+1 will be

(13) $$z_{k+1}^{s}=z_{k}^{s}-d_{k+1}^{s},$$

where $d_{k+1}^{s}$ is the distance to move down at time k+1. It can be seen that, given node depth at time k+1, the FIM of target location is a function of movement distance vector $D_{k+1}={\left[d_{k+1}^{1},\cdots ,d_{k+1}^{N_{s}}\right]}^{T}$.

In this paper, the problem we address is how to determine nodes’ optimal depth to maximize determinant of FIM under the constraints on movement of nodes. Based on the relation between FIM and node depth, the optimization problem can be formulated as:

(14) $$\begin{array}{c} \min_{D_{k+1}}-\det\left(J_{k+1}\left(D_{k+1}\right)\right),\\ \mathrm{subject}\;\mathrm{to}\;\;\;\;d_{min}\leq d_{k+1}^{s}\leq d_{max},\\ d_{k+1}^{s}\in D_{k+1},\;\;\;\;s\in \left\{1,\cdots ,N_{s}\right\}, \end{array}$$

where det(·) denotes the determinant calculation of matrix, $d_{min}$ and $d_{max}$ are the lower and upper bound on moving distance, which is a dynamic variable according to target location.

So far, we have converted the node depth adjustment to an optimization problem. We need to find a solution to the dynamic optimization problem. It is extremely difficult to find a global optimal analytical solution. The computation will be too large if we discretize the moving range and calculate the determinant of FIM with an exhaustive search. It is necessary to find a method applied to this problem with proper computation. The results will be presented in the next section.

## 5. Improved Harmony Search for Node Depth Adjustment

As mentioned before, this paper adopts the harmony search algorithm (HS) and modifies it to improve searching speed. HS is a meta-heuristic algorithm that mimics the improvisation process of music players. In recent years, HS has been successfully used in a wide variety of optimization problems. Compared with traditional optimization algorithms, HS requires fewer mathematical computations and does not require setting initial values of decision variables. In addition, a new vector is generated after considering all existing vectors, which is different from genetic algorithms (GAs). These features increase the flexibility of HS and lead to better performance. This section presents how to modify the HS algorithm to improve tracking accuracy with adjusting node depth to the optimal depth.

The HS algorithm begins with a population of vectors, which is the Harmony Memory (HM). Each of these vectors stands for a combination of all activated sensors’ moving distance. In the initial step, these vectors are randomly generated within the moving range. Then, the evolving process of HS will be started with the improvisation process of the new solution vector. At each iteration, a new vector can be generated in two ways. The new solution vector may be selected from HM solutions or generated randomly as initialization to diversify HM. Then, we will use pitch adjustment to locally adjust the new solution vector. The new vector will be compared with the worst vector in HM, the better one will be left in HM, and another will be removed. After reaching the maximum iteration, the best vector will be selected as a final solution. In addition, to improve searching speed, we adjust the probability of a new harmony to be generated as increasing as the distance to the target decreases. The reason is that some vectors far from targets may be not that useful, and it will waste many resources and much time to adjust these vectors. In addition, the pitch adjustment rate and bandwidth dynamically changes during iteration to improve performance. Furthermore, the objective function is one of the important factors to all optimization problems, and a solution with the best function value is selected as the final result. As shown before, the determinant of FIM in Equation (14) is used as an objective function to evaluate solution vectors. All details of the improved HS based depth adjustment method are presented in the following five steps.

### 5.1. Initialization of Parameters and the Problem

As mentioned before, the optimization problem is shown as Equation (14), where $\det\left(J_{k+1}\left(D_{k+1}\right)\right)$ is the objective function; all $N_{s}$ nodes’ movement distance vector $D_{k+1}$ is the set of all decision variables. In addition, the parameters of HS algorithm will be specified in this step. The parameters are as follows:

1. Harmony Memory Size (HMS); it means the number of solution vectors in HM;
2. Harmony Memory Considering Rate (HMCR); it determines selecting a vector from HM or randomly generating a new vector;
3. Pitch Adjusting Rate (PAR); it is the probability to pitch adjust a new vector;
4. Bandwidth (BW); it is used to adjust the new vector to improve the performance of HS;
5. Number of iterations (NI); it is the maximum number of iterations to be used as a stopping criterion.

### 5.2. Initialization of Harmony Memory

The harmony memory (HM) in Equation (15) is a matrix with the length of HMS and width of $N_{s}$ that stores solution vectors:

(15) $$\mathrm{HM}=\left[\begin{array}{ccccc} d_{1}^{1} & d_{2}^{1} & \cdots & d_{N_{s}-1}^{1} & d_{N_{s}}^{1}\\ d_{1}^{2} & d_{2}^{2} & \cdots & d_{N_{s}-1}^{2} & d_{N_{s}}^{2}\\ \vdots & \vdots & \vdots & \vdots & \vdots \\ d_{1}^{HMS-1} & d_{2}^{HMS-1} & \cdots & d_{N_{s}-1}^{HMS-1} & d_{N_{s}}^{HMS-1}\\ d_{1}^{HMS} & d_{2}^{HMS} & \cdots & d_{N_{s}-1}^{HMS} & d_{N_{s}}^{HMS} \end{array}\right].$$

For traditional HM algorithms, solution vectors in this step are randomly generated within moving distance range. However, this is not efficient because those nodes far from target are very likely to provide little information about target. In this step, we modify the probability of generating a new harmony. Instead of using a random generation probability, we make generation probability increase with distance to target decreasing. We assume that the probability is linearly correlated to moving distance, and the scale factor is $p_{0}$. Thus, within the moving range, we will have the cumulative distribution function as follows:

(16) $$\begin{array}{c} \int_{d_{min}}^{d_{max}}p\left(l\right)dl=1,\\ p\left(l\right)=p_{0}l, \end{array}$$

where *l* is the moving distance, $d_{min}$ and $d_{max}$ are the lower and upper bounds of moving distance range; we always have $d_{min}=0$, which means that nodes will not move, and $d_{max}$ is dynamically set to enable nodes moving to the same depth as predicted positions of nodes. Thus, we can solve Equation (16) and get the value of $p_{0}$ and generation probability p(l) of moving distance *l*:

(17) $$\begin{array}{c} p_{0}=\frac{2}{d_{max}^{2}-d_{min}^{2}},\\ p\left(l\right)=\frac{2l}{d_{max}^{2}-d_{min}^{2}}. \end{array}$$

With this generation probability method, we have initial solution vectors to fill the HM matrix. The moving distance leading node close to the target will provide more useful information to track the target. Therefore, this method will improve searching speed and accuracy. It should be noted that this generation probability method is not only used in the initial step, but it is also used in the third step, in which we may need to generate a vector within range.

After the HM is generated with different moving distance combinations of activated nodes in each solution vector, the quality of each is evaluated with estimation accuracy, and it is calculated with the proposed objective function in Equation (14). As shown in Equation (14), the smaller the value is, the better the vector is.

### 5.3. Improvise a New Harmony

To find the optimal solution vector, we need to improvise a new harmony during each iteration. The new harmony vector is also a combination of all activated sensors’ moving distance . As shown in Equation (18), the new vector can be generated in two ways. To determine the way to generate new harmony, we need generate a random number within [0, 1], and compare it with the probability of HCMR. If the number is less than HMCR, the components of new harmony will get the value from the same column in HM. If the generated random number drop in the probability of 1−HMCR, the new components will be randomly generated within moving range in the same way as in Step 2. It should be noted that generation way of each component is determined respectively.

(18) $$d_{i}^{\prime }\leftarrow \left\{\begin{array}{c} d_{i}^{\prime }\in \left\{d_{i}^{1},d_{i}^{2},\ldots ,d_{i}^{HMS}\right\}\;\text{with probability HMCR},\\ d_{i}^{\prime }\in \left[d_{min},d_{max}\right]\;\;\;\text{with probability 1-HMCR}. \end{array}\right.$$

For those components selected from HM, they need to be examined to determine if it would be pitch adjusted. Similar to HMCR, PAR is used to determine the pitch adjustment. If random number is less than PAR, we will pitch adjust the component. The pitch adjustment decision process is as follows:

(19) $$\left\{\begin{array}{c} Yes\;\;\text{with probability PAR},\\ No\;\;\text{with probability 1-PAR}. \end{array}\right.$$

If a component needs to be pitch adjusted, the bandwidth BW and a random number α between [0, 1] are used to adjust it as follows:

(20) $$d_{i}^{\prime }=d_{i}^{\prime }\pm \alpha \times \mathrm{BW}.$$

For the traditional HS algorithm, HCMR, PAR and BW are static parameters in the iteration process. However, it would lead to local optimum easily. Therefore, it is important to dynamically update these parameters with increasing of iterations. The detail equations in generation gn are as follows:

(21) $$\left\{\begin{array}{c} \mathrm{HCMR}(\mathrm{gn})={\mathrm{HCMR}}_{min}\times \exp\left(\frac{\mathrm{gn}\times \ln(\frac{{\mathrm{HCMR}}_{max}}{{\mathrm{HCMR}}_{min}})}{\mathrm{NI}}\right),\\ \mathrm{PAR}(\mathrm{gn})={\mathrm{PAR}}_{min}+\frac{{\mathrm{PAR}}_{min}-{\mathrm{PAR}}_{min}}{\mathrm{NI}}\times \mathrm{gn},\\ \mathrm{BW}(\mathrm{gn})={\mathrm{BW}}_{max}\times \exp\left(\frac{\mathrm{gn}\times \ln(\frac{{\mathrm{BW}}_{max}}{{\mathrm{BW}}_{min}})}{\mathrm{NI}}\right), \end{array}\right.$$

where the subscript max and min denote maximum and minimum value of corresponding parameters.

### 5.4. Update Harmony Memory

As shown in step 1, each harmony vector is evaluated using objective function and the value is stored in HM matrix. After generating a new vector, we also calculate its quality value using Equation (14), and compare it with the worst harmony vector in terms of the objective function. Then, the better one will be stored in the HM matrix, and another one will be eliminated. In this way, harmony vectors in the HM matrix can be improved at each iteration.

### 5.5. Check Stop Criterion

The whole process will be performed iteratively, and it will keep improvising new harmony vectors and update the HM matrix until the maximum iteration number (NI) is reached. Then, the harmony vector with the best value of objective function is selected as the final solution vector. It means that this vector will be used to adjust nodes to the optimal depth to improve tracking accuracy in each time interval.

So far, we have finished the improved HS algorithm to determine optimal depth of activated nodes. In this method, the determinant of FIM at each interval is maximized to improve tracking accuracy. The detail pseudo code of the improved HS algorithm for depth adjustment is listed as Algorithm 1.

**Algorithm 1** Node depth adjustment based target tracking scheme using improved harmony search  1:**1. Initialization:**  2:**if** k=0 **then**  3: Particle Initialization:  4: **for** i=1,2,…,N **do**  5:  draw particle $x_{k}^{i}$ from the prior of target state $p(x_{0})$;  6: **end for**  7:**end if**  8:**2. Tracking process:**  9:**for** k=1,2,… **do**10: **for** i=1,2,…,N **do**11:  sample $x_{k+1}^{i}∼p\left(x_{k+1}|x_{k}^{i}\right)$;12: **end for**13: {Start adjusting nodes’ depth}:14: Define parameters: HMS, HMCR, PAR, BW, NI, $d_{min}$, $d_{max}$15: Initialize new harmony vectors using Equations (16) and (17)16: **while** gn≤NI **do**17:  Update parameters using Equation (21)18:  Check improvising a new harmony vector using Equation (18)19:  **if** rand∈(0,1)≤HMCR **then**20:   Select from HM21:   Check pitch adjustment using Equation (19)22:   **if** rand∈(0,1)≤PAR **then**23:    pitch adjust using Equation (20)24:   **end if**25:  **else**26:   Generate from moving range $\left[d_{min},d_{max}\right]$ using Equations (16) and (17)27:  **end if**28:  Update Harmony Memory29:  Calculate objective function value using Equation (14)30:  Compare new vector to the worst one in HM, and keep the better one.31: **end while**32: Select the best vector as optimal depth solution33: {END adjusting nodes’ depth}34: Get the measurements from depth adjusted nodes and estimation using Particle Filter.35:**end for**

## 6. Simulations

In this section, we present the simulation results of underwater target tracking with our node depth adjustment method. Our node depth adjustment is based on the improved Harmony Search algorithm presented in Section 4. In addition, we also present the results of nodes keeping static as a comparison and analyze the effect of some parameters. Furthermore, to evaluate our method well, we present the convergence process of objective function value via our improved Harmony Search and traditional Harmony Search.

**Figure 2:**
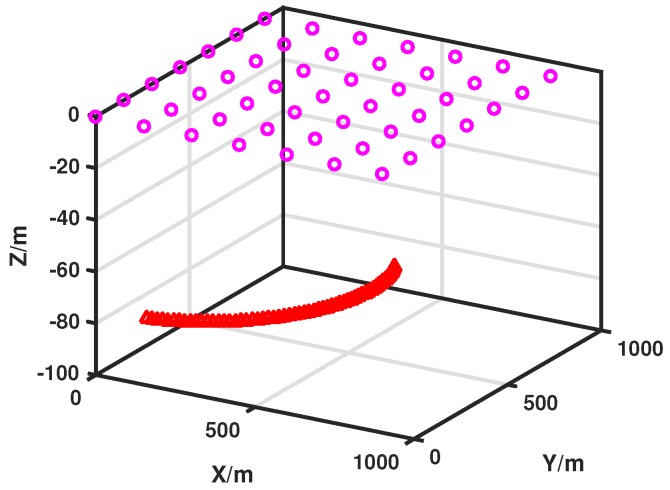
Simulation scenario (the purple circles stand for sensor nodes. They are deployed at water surface at first, the red triangle stands for moving target, and it moves in a plane underwater).

We consider a 1000×1000 region where sensors are uniformly deployed on the sea surface. The target is moving in a plane at a known depth *h*. The distance between adjacent nodes is dis. The simulation scenario is shown in Figure 2. In the simulation, the number of selected nodes at each interval is $N_{s}=3$; the actual initial state of target is $x_{0}={\left[\begin{array}{cccc} 100 & 15 & 100 & 3 \end{array}\right]}^{T}$; the initial estimation of target is ${\hat{x}}_{0}={\left[\begin{array}{cccc} 95 & 15 & 105 & 3 \end{array}\right]}^{T}$; the initial covariance matrix is $P_{0}=10\times I_{4\times 4}$; the standard deviation of measurement noise is assumed to be constant as r=5; the standard deviation of process noise is q=1; the sampling interval is T=1s; the particle number is N=1000; the Monte Carlo (MC) simulation runs MC=100 and the simulation is run with Matlab R2016a (MathWorks, Inc., Natick, MA, USA), in a computer with CPU i7-7700HQ.

To indicate the accuracy of target tracking, we adopt root mean square error (RMSE) to measure the tracking performance:

(22) $$\epsilon \left(k\right)=\sqrt{\sum_{i=1}^{MC}\left(\frac{{\left(x_{k}^{i,1}-{\hat{x}}_{k}^{i,1}\right)}^{2}+{\left(x_{k}^{i,3}-{\hat{x}}_{k}^{i,3}\right)}^{2}}{MC}\right)},$$

where $\left(x_{k}^{i,1},x_{k}^{i,3}\right)$ and $\left({\hat{x}}_{k}^{i,1},{\hat{x}}_{k}^{i,3}\right)$ are true and estimated locations of the target at time *k* in the *i*th simulation, respectively.

**Figure 3:**
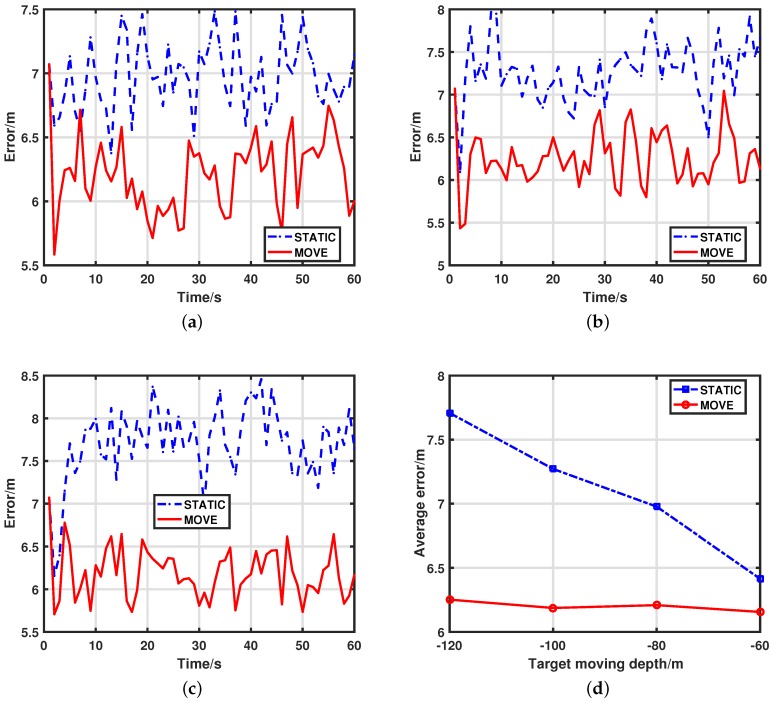
The tracking error comparison between MOVE and STATIC with different moving depths. (**a**) tracking errors for MOVE and STATIC with moving depth h=−80 (over 100 MC runs); (**b**) tracking errors for MOVE and STATIC with moving depth h=−100 (over 100 MC runs); (**c**) tracking errors for MOVE and STATIC with moving depth h=−120 (over 100 MC runs); (**d**) average tracking errors for MOVE and STATIC with different moving depths (over 100 MC runs).

Simulation results of our scheme and keeping nodes static are presented with different target depths. We define depth of the sea surface as 0. Then, the target depth is h={−120,−100,−80,−60}. The distance between adjacent nodes is dis=120. For simplicity, we use MOVE to denote our scheme and use STATIC to denote the scheme that keeps nodes static at the sea surface. The results with different target depths are shown in Figure 3. It can be seen that our MOVE scheme can improve much performance compared to the STATIC scheme. This is because the MOVE scheme always adjusts activated nodes to the optimal depth by maximizing the determinant of FIM, thus providing more useful information and leading to better performance. Because the STATIC scheme keeps nodes static at the sea surface, it can not get enough information about targets and achieve tracking accuracy as well as MOVE. It is verified that our MOVE can improve tracking performance a lot.

The corresponding average tracking errors of the two schemes with moving depth h={−60,−80,−100,−120} are shown in Figure 3d and Table 1. It is obvious that tracking error increases with declining of moving depth *h*. It shows that, as moving depth decreases, our MOVE scheme always produces good results, while the errors of the STATIC scheme increase a lot. The main reason for this is that, when sensors are far from targets, they can not get enough information of targets at an unideal position. However, with our scheme, we can always adjust nodes to optimal depth to provide as good measurements as possible. Hence. the MOVE scheme can improve the tracking performance a lot especially when nodes are far from targets.

It should be noted that different node numbers have an effect on tracking performance. With more nodes, we can get more information and achieve a better tracking performance, but it may lead to higher energy consumption. How to find a balanced method between energy consumption and tracking error via different node number is an important problem. However, it is not the main concern in this paper; thus, we always wake up three nodes during the tracking process. We will study the adaptive method to determine node number in future work. In addition, our method is not limited to the water surface network. The reason we use such a network is that it is more practical and cheaper than 3D networks.

**Figure 4:**
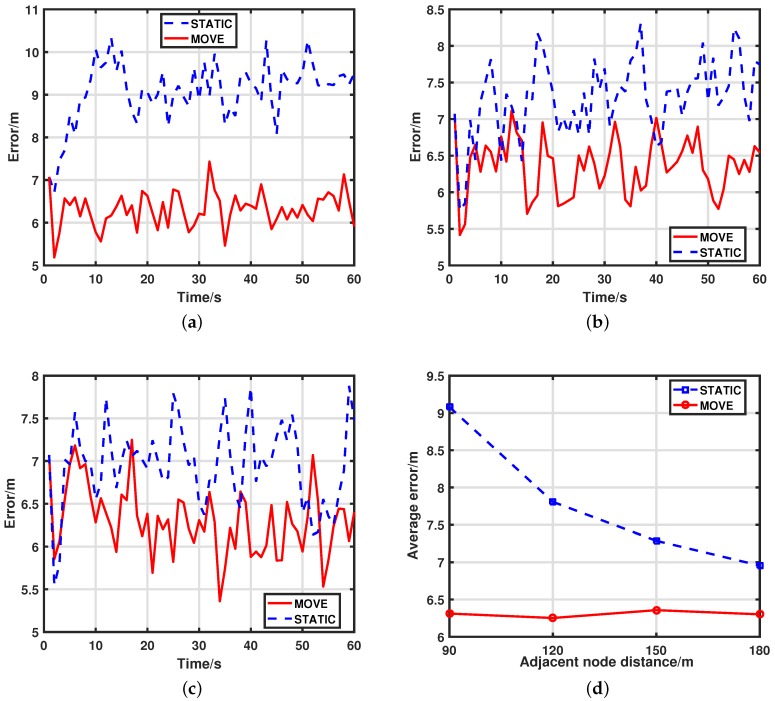
The tracking error comparison between MOVE and STATIC with different adjacent node distance. (**a**) tracking errors for MOVE and STATIC with adjacent node distance dis=90 (over 100 MC runs); (**b**) tracking errors for MOVE and STATIC with adjacent node distance dis=150 (over 100 MC runs); (**c**) tracking errors for MOVE and STATIC with adjacent node distance dis=180 (over 100 MC runs); (**d**) average tracking errors for MOVE and STATIC with different adjacent node distance (over 100 MC runs).

Then, we compare the effect with different node density in UWSNs. The simulation results of MOVE and STATIC with node distance dis={90,150,180} are plotted in Figure 4 (results of dis=120 have been presented in Figure 3c). It is not surprising that the MOVE scheme always performs better than STATIC. As shown in Figure 3d and Table 2, our MOVE scheme can maintain good performance no matter whether UWSNs are sparse or dense due to the fact that it can adjust nodes to the optimal depth at all times. On the contrary, the node density has a big effect on tracking accuracy of the STATIC scheme. The reason is that when nodes are sparsely deployed in the network, the activated nodes are far from each other, and they can measure the targets in different positions and angles. However, in densely deployed networks, the activated nodes are close to each other. Thus, a lot of information provided by their measurements is overlapping. As shown in the results, our MOVE scheme is a good solution to this problem.

**Figure 5:**
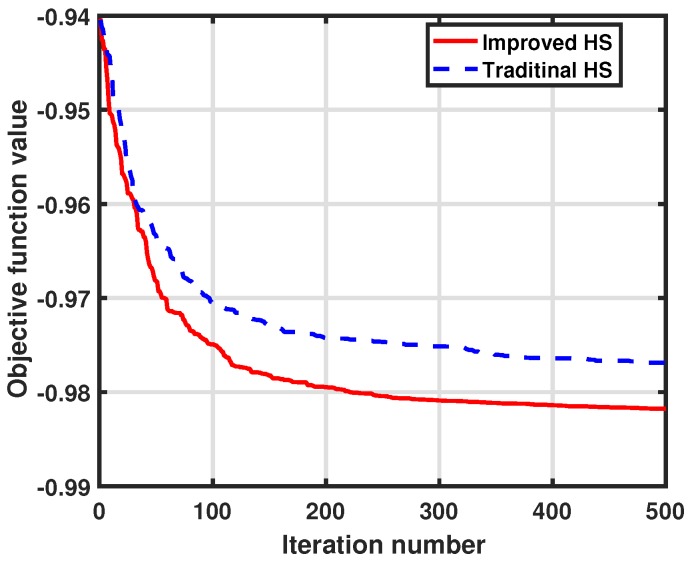
Convergence progress of objective function value with different HS algorithms.

Furthermore, the searching performance of our improved HS algorithm is presented in Figure 5. We use the convergence process of objective function value to show the searching performance of the optimization method. In addition, the convergence process of traditional HS algorithms is presented as a comparison. In this simulation, we show the 500 iterations’ convergence process of objective function value via two methods at the same time interval k=25, and the results are the averaged value after 100 Monte Carlo simulations.

It is obvious that our improved HS algorithm can achieve faster searching speed and better convergence value. For our improved method, objective value converges to −0.9818, while the value with the traditional method only converges to −0.9769. In addition, our improved HS algorithm needs less iteration to achieve the same objective value, and can achieve a better value finally. The reason is that, in our improved HS algorithm, the generating probability of a new harmony vector has been modified to be linearly correlated to moving distance. Thus, it has a higher probability to move to a better depth. Hence, our improved HS algorithm is an efficient method for node depth adjustment.

## 7. Conclusions

This paper proposed a node depth adjustment based scheme for target tracking in UWSNs. A novel node depth adjustment problem is formulated under the framework of an optimization problem to achieve more accuracy tracking performance. To solve this problem, an improved Harmony Search algorithm is designed to efficiently determine the optimal depth of activated nodes. It is the first time to use node depth adjustment to improve tracking accuracy. To verify the effectiveness of our scheme, simulations’ results are presented and show that our scheme can achieve better tracking performance than existing schemes. Further work will consider the balance between tracking accuracy and energy consumption in node depth adjustment and extend the results to multiple targets tracking. In addition, due to complex underwater environments, node location errors and complex measurement models are also important problems to be solved in the future.

## Figures and Tables

**Table 1:** Average tracking errors with different moving depth *h*.

Schemes	*h* = −120	*h* = −100	*h* = −80	*h* = −60
STATIC	7.807	7.271	6.977	6.415
MOVE	6.252	6.187	6.209	6.156
Improvement	19.92%	14.91%	11.01%	4.04%

**Table 2:** Average tracking errors for MOVE and STATIC with different adjacent node distance dis *L*.

Schemes	*dis* = 90	*dis* = 120	*dis* = 150	*dis* = 180
STATIC	9.079	7.807	7.287	6.955
MOVE	6.311	6.252	6.357	6.301
Improvement	30.49%	19.92%	12.76%	9.40%
